# Epidemiological, clinical, and molecular characterization of Cuban families with spinocerebellar ataxia type 3/Machado-Joseph disease

**DOI:** 10.1186/s40673-015-0020-4

**Published:** 2015-02-21

**Authors:** Yanetza González-Zaldívar, Yaimeé Vázquez-Mojena, José M Laffita-Mesa, Luis E Almaguer-Mederos, Roberto Rodríguez-Labrada, Gilberto Sánchez-Cruz, Raúl Aguilera-Rodríguez, Tania Cruz-Mariño, Nalia Canales-Ochoa, Patrick MacLeod, Luis Velázquez-Pérez

**Affiliations:** Centre for the Research and Rehabilitation of Hereditary Ataxias (CIRAH), Libertad Street 26, Holguín, Postal code 80100 Cuba; Division of Medical Genetics, Department of Pathology, Laboratory Medicine and Medical Genetics, Victoria General Hospital, Victoria, Canada

**Keywords:** Spinocerebellar ataxia type 3, Machado-Joseph Disease, SCA3/MJD, CAG repeats

## Abstract

**Background:**

Spinocerebellar Ataxia Type 3/Machado-Joseph Disease (SCA3/MJD) is a hereditary neurodegenerative disorder resulting from the expansion of CAG repeats in the *ATXN3* gene. It is the most common autosomal dominant ataxia in the world, but its frequency prevalence in Cuba remains uncertain. We undertook a national study in order to characterize the *ATXN3* gene and to determine the prevalence of SCA3/MJD in Cuba.

**Results:**

Twenty-two individuals belonging to 8 non-related families were identified as carriers of an expanded *ATXN3* allele. The affected families come from the central and western region of the country. Ataxia of gait was the initial symptom in all of the cases. The normal alleles ranged between 14 and 33 CAG repeats while the expanded ones ranged from 63 to 77 repeats. The mean age at onset was 40 ± 9 years and significantly correlated with the number of CAG repeats in the expanded alleles.

**Conclusions:**

This disorder was identified as the second most common form of spinocerebellar ataxia (SCA) in Cuba based on molecular testing, and showing a different geographical distribution from that of SCA2. This research constitutes the first clinical and molecular characterization of Cuban SCA3 families, opening the way for the implementation of predictive diagnosis for at risk family members.

## Background

Spinocerebellar Ataxia Type 3 (SCA3) also known as Machado-Joseph Disease (MJD) is a polyglutamine neurodegenerative disorder caused by the expansion of a (CAG)_n_ in the *ATXN3* gene. Normal alleles range from 11 to 44 CAG repeats, whereas pathogenic expansions range from 61 to 87 CAGs [1].

A variety of clinical features can be observed in subjects carrying ≥52 CAG units [2-4]. The first linkage studies were facilitated by the identification of founders with Portuguese-Azorean ancestry [5].

SCA3 is characterized by cerebellar ataxia, ocular rotatory muscles palsy, gaze-evoked nystagmus, pyramidal and extrapyramidal signs, as well as sleep disorders. The age of onset of SCA3/MJD is variable, but most commonly in the second to fifth decade. Taking into consideration the variability of the age at onset and clinical manifestations, five subtypes of SCA3/MJD can be recognized [6-8].

At worldwide, the disease is the most common form of autosomal dominant ataxia [3,9,10] and several studies have been reported [11]. Nevertheless, in Cuba there is little information on the epidemiological, clinical and molecular features of the SCA3. A national survey conducted to establish the epidemiological profile of inherited ataxias in this island identified a reduced frequency of *ATXN3* gene in the Cuban population with autosomal dominant cerebellar ataxias, whereas the molecular and clinical data were not provided [12]. Moreover, the variation of the CAG repeats in the general population may be important to predict the prevalence of chromosomes predisposed to undergo CAG expansions and consequently cases do to *de novo* mutations.

Therefore, a clinical and molecular genetic study of a cohort of SCA3 families was developed to determine the prevalence and distribution of MJD/SCA3 in the Cuba. The normal variation of CAG repeats and their unstable behavior were also evaluated.

## Results

### Epidemiological findings

**Figure 1:**
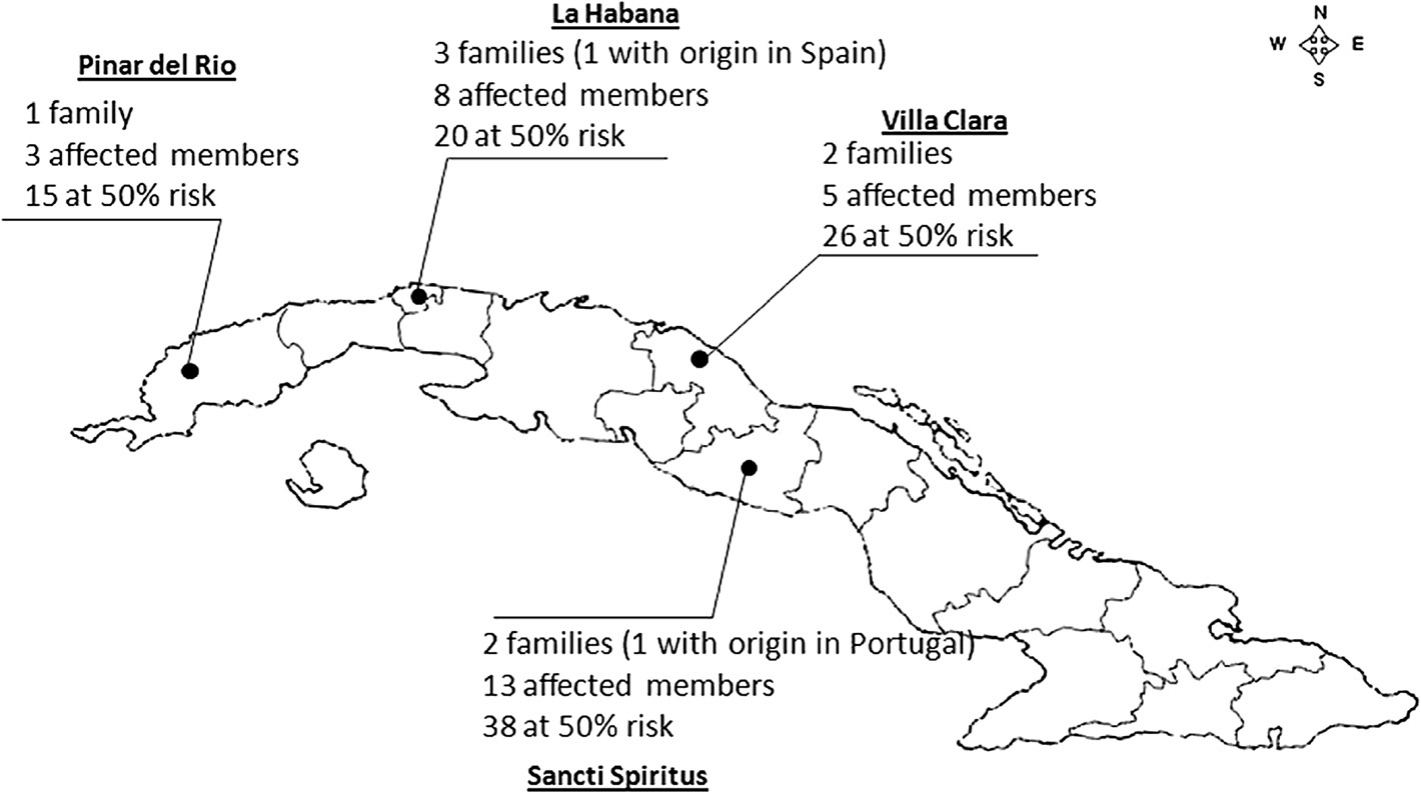
**Distribution of families affected by SCA3/MJD in Cuba.**

Mean age of the included patients was 45.47 years (range 14–73, Standard deviation (SD) 13.62) whereas the mean of available ages at onset was 40.64 years (range 13–64, SD 11.92). Age at onset was available for 18 of the 22 molecularly confirmed cases, with a mean of 40 ± 9 years (range 21–55 years) and an inverse correlation with the expanded CAG repeats (r = −0.81; p = 0.00005) (Figure 1).

**Figure 2:**
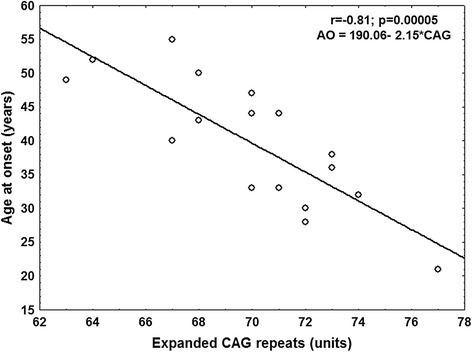
**Age of onset and CAG correlation: Significant inverse correlation between age of onset and expanded CAG repeats.** AO: Age at onset; CAG: Expanded CAG repeats.

The SCA3 mutation was detected in 22 out 136 individuals (16%), who belonged to 8 apparently unrelated families (three families with two affected members, two with four affected members, two families with one affected member and one family with six affected members). In these families, the genealogical information indicated the existence of 55 affected individuals and 99 first degree at-risk relatives. Also, the recognized genealogical origins in two distinct families were from Portugal and Spain. Among affected individuals, only 29 cases were still alive in Cuba. This suggests a national SCA3 prevalence of 0.26 cases/100 000 inhabitants and a frequency of 4.1% among the dominant ataxias in Cuba. Analysis of the geographic location of affected families revealed an origin at the central-western region of the country (Figure 2).

### Clinical features in SCA3/MJD patients

All SCA3 patients showed gait ataxia as initial symptom of the disease. Almost all of them retained gait autonomy with the exception of five patients (22.7%) who required external support and four (18.2%) who used a wheelchair. Other cerebellar features were dysdiadochokinesia (16 out 22, 73%), cerebellar dysarthria (15 out 22, 68%) dysmetria (15 out 22, 68%) and kinetic tremor (14 out 22, 64%). Nine patients suffered from dysphagia (41%). Pathological nystagmus was the most important oculomotor sign (17 out 22, 77%), followed by horizontal saccade slowing in 7 out 22 cases (32%).

Among the non-cerebellar features, painful muscle cramps were the most common symptoms appearing in 18 out 22 subjects (82%), followed by hypoesthesia, paresthesia or hypopallesthesia in 13 out 22 cases (59%). Facial atrophy was observed in nine out 22 (41%) subjects. Pyramidal features (hyperreflexia, Babinski sign and fasciculations) were present in three patients (10%) with more than 20 years of disease duration. Two patients referred manifestations of depression (9%) and fatigue was reported by one case (4.5%). Four subjects (18%) manifested spasticity, resembling a phenotype similar to hereditary spastic paraplegia with cerebellar ataxia. Craniocervical dystonia was present in one patient, who carried the largest CAG repeat and earliest age at onset. Eyelid retraction was not observed. Neither signs of Parkinsonism nor chorea were detected.

### Molecular analysis of the *ATXN3* gene

**Figure 3:**
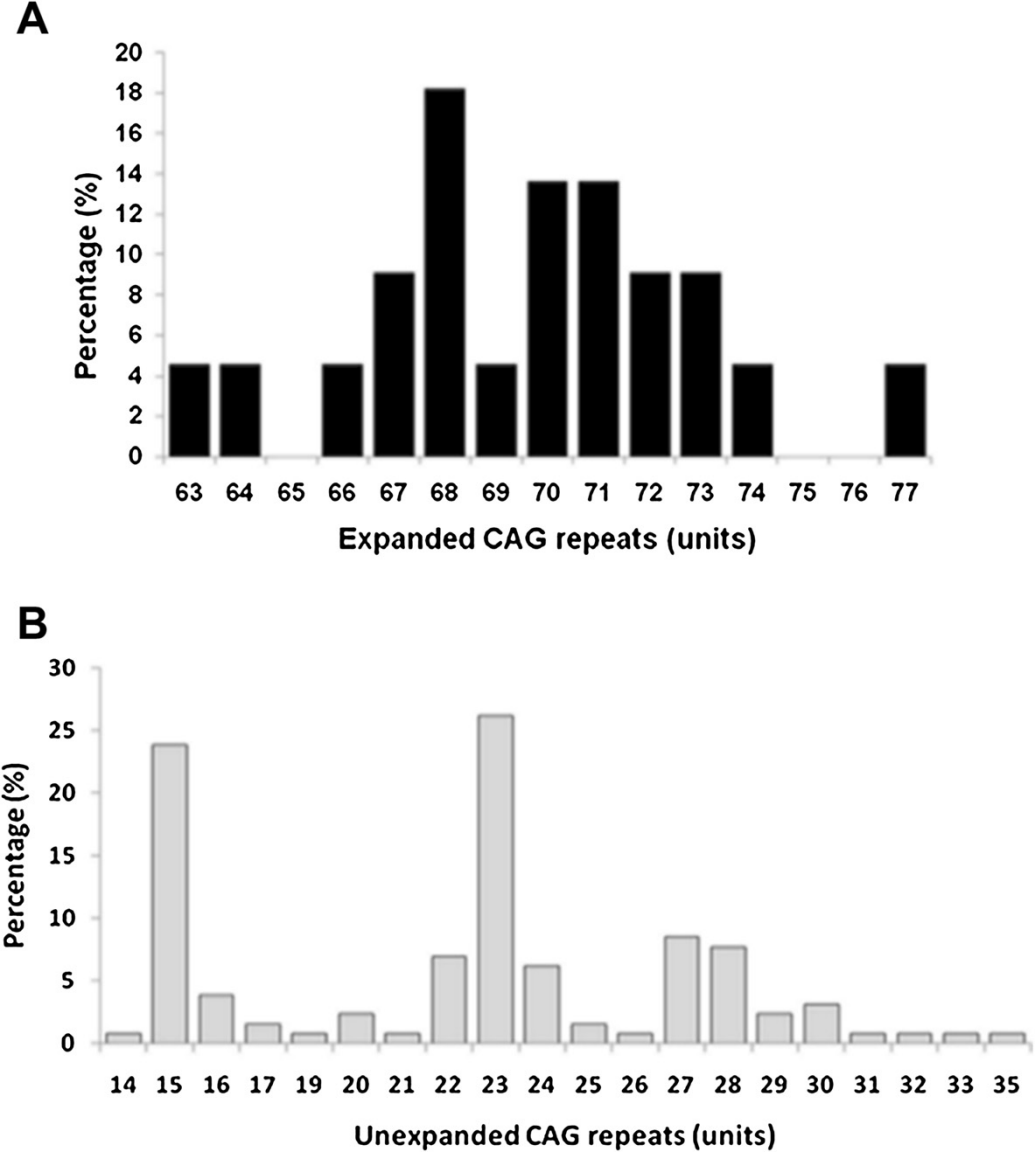
**Frequency distribution for expanded** ***ATXN3*** **alleles from the 22 confirmed SCA3 patients (A) and for the unexpanded** ***ATXN3*** **alleles from all patients and the 65 healthy controls (B).**

Expanded alleles varied from 63 to 77 CAG repeats (mean 69.6, SD 3.29), following a normal distribution (K-S D = 0.01; p > 0.20). The skew of the distribution was 0.03 ± 0.49. Thirteen distinct allele’s classes were detected. Alleles with 68 CAG repeats were the most frequent (18.2%) (Figure 3A). All expanded CAG repeats classified as alleles with full penetrance (>52 CAG).

Up to 20 transmissions of the expanded CAG alleles were observed (15 paternal and 5 maternal). The CAG size of the expanded alleles in patients with paternal inheritance (mean 69.2, SD 3.33) and maternal inheritance (mean 71.6, SD 4.06) were not statistically different (p = 0.420). CAG size transmission was analysed in only three parent–child pairs; without expansions or contractions.

All control individuals carried non-expanded CAG repeats. Twenty allelic classes were found, ranging from 14 to 35 CAG repeats (mean: 22.0, SD 5.17) (Figure 3B). Alleles with 23 (26.2 %) and 15 (23.8 %) CAG repeats were the most frequent. The allele’s distribution in this group was bimodal and not normal (K-S D = 0.17; p < 0.01), showing a negative skew (−0.007 ± 0.21).

## Discussion

Spinocerebellar Ataxia Type 3 is the commonest autosomal dominant inherited spinocerebellar ataxia worldwide, with variable prevalence according to ethnic background [11]. The present paper showed the epidemiological, clinical and molecular characterization of SCA3 in Cuba, resulting from the nationwide epidemiological survey for SCAs [12]. These findings identify SCA3 as the second most frequent molecularly confirmed autosomal dominant cerebellar ataxia in Cuba, only surpassed by SCA2, but more prevalent than SCA7 (0.10%), and SCA1 (0.05%) [13].

The frequency of SCA3 is lower in Cuba than reported in Brazil (72%) [14], Portugal (58%) [15], China (48%) [16], Japan (28%), Germany (42%) and Spain (15%) [6,11], but is similar to those in India (5%), a country with a relatively higher frequency of SCA2 mutations [17]. Although our genealogical data revealed the origin of two Cuban kindred from populations with higher SCA3 frequencies, further haplotype studies are needed to determine the ancestral origin of SCA3 mutation in the Cuban population, since previous analyses of *ATXN3* gene polymorphisms have supported evidences of independent and multiple origin of SCA3 in different populations [5].

Our findings confirmed the progressive, multisystemic and pleomorphic nature of SCA3 [6]. The main clinical features of Cuban SCA3 patients were in agreement with previous reports. [6,7,18,19]. Pathological nystagmus is the most frequent oculomotor alteration of SCA3 patients [19] being present in 90% of the cases. In the present paper we confirmed the higher frequency of this feature, suggesting the marked involvement of cerebellar oculomotor circuitry in these patients, whereas the relatively small lower frequency of saccade slowing suggests the less involvement of pontine nuclei compared to SCA2 and SCA7, which has been previously confirmed by neuropathological findings [20].

Non-cerebellar manifestations are common features of SCA3 patients [21], which were confirmed in the Cuban SCA3 population. Among them, cramps were the most prevalent, with a frequency similar to that reported by Kanai and co-workers in 2003 [22], who identified an altered motor axonal excitability as the physiopathological origin of this feature. These findings have important implications for the management of patients due to the intensity and frequency of cramps in SCA3. Some patients have reported improvement using carbamazepine and mexiletine [22,23], which can be applied in Cuban SCA3 subjects as symptomatic therapeutical options.

Other frequent non-cerebellar features observed in this cohort were the clinical signs of peripheral neuropathy, which was observed as frequent as in previous reports [24,25] and suggest the involvement of peripheral nervous system in SCA3, as has been confirmed previously by neurophysiological studies [24-26]. As the peripheral signs are characteristic for the Type III SCA3 subtype, our findings suggest the higher prevalence of this phenotype in the Cuban population with SCA3 symptoms. Depression was the most common psychiatric feature in agreement with other authors [27,28].

In our cohort, the percentage of SCA3 patients exhibiting pyramidal signs is similar to observed by Jardim and co-workers in 2001 [19] in a large cohort of Brazilian SCA3 patients. Also, it is known that SCA3 patients present cerebellar ataxia associated spasticity, resembling the hereditary spastic paraplegia phenotype. Wang and co-workers identified CAG expansions in the *ATXN3* gene of six Chinese subjects with hereditary spastic paraplegias (HSP) [8]. Our findings of SCA3 Cuban patients with a phenotype similar to hereditary spastic paraplegia are in agreement with this report, supporting a consideration to analyse the *ATXN3* gene in patients clinically diagnosed as HSP. In SCA3, dystonia is usually associated to early onset of the disease and larger CAG expansions [14,21], which was confirmed in the present study because the only patient exhibiting dystonia had the largest CAG expansion and earliest age at onset, belonging to the Type I SCA3 subtype [7]. Although eyelid retraction and signs of Parkinsonism have been observed in SCA3 patients, these features were not observed in the Cuban cohort.

Our data confirmed a significant correlation between expanded CAG repeats and age at onset, demonstrating that the expanded CAG size in the *ATXN3* gene is responsible of the approximately 80% of the age at onset variability in the patients, which suggests the existence of other genetic and non-genetic factors influencing the SCA3 age at onset. A recent study carried out in 12 Cuban SCA3 patients demonstrated that the hypermethylation of the *ATXN2* gene correlated with earlier onset of SCA3 symptoms, which identify this epigenetic feature as a modifying factor of SCA3 [29].

The effect of gender on the intergenerational instability of the expanded CAG repeats was analysed. Paternal mutant alleles are slightly more unstable than maternal ones thus they are more prone to expand or to contract when transmitted to the next generation [30]. Although over one half of parent–child transmissions in the MJD repeats are unstable [7], in our sample, CAG instability was not detected. However, age at onset anticipation (−11 years) was found in a parent–child transmission without CAG repeat expansion as formerly documented [31]. Nevertheless, further analyses in larger cohorts are recommended.

The range of normal and expanded alleles found in our study is similar to those reported in other populations [31,32]. Their distribution showed well established limits between normal and expanded repetitions without reduced penetrance alleles, making it possible to confirm or exclude the molecular diagnosis. In accordance with other reports, the distribution of normal alleles showed a bimodal pattern [32,33], which is similar to that found by Lima and co-workers in a Portuguese population [32]. In both distributions, alleles with 23 repeats were the most frequent.

It had been suggested that large normal alleles (>27 repeats) may constitute a reservoir for the emergence of expanded alleles, since both share the same haplotypes [33]. Although the SCA3 frequency in our study is lower than in other studies, the observation of a frequency of large normal alleles of 0.25 deserves further evaluation of predisposed haplogroups. A recent study revealed that longer *ATXN3* alleles were associated with earlier ages at onset for SCA2 in a Brazilian population of SCAs patients [34]. As SCA2 is the most common subtype of SCAs in Cuba this finding needs to be confirmed in the homogeneous population of SCA2 patients living in Cuba.

## Conclusion

In conclusions, SCA3 was identified as the second most common recognized spinocerebellar ataxia in the Cuba showing different epidemiological characteristics to SCA2. This research reports the first clinical and molecular characterization of Cuban SCA3 families, providing the knowledge necessary for the establishment of predictive and confirmatory diagnosis and the possibility of clinical trials for at risk family members. Haplotypes studies have been helpful in diverse populations [35] and we plan similar studies to determine the origin of the SCA3 founder mutation in the Cuban population.

## Methods

### Study design

This study represents a prospective epidemiological survey of SCA3 in the Cuban population during 2007–2013 as part of a nationwide epidemiological study of Hereditary Ataxias in Cuba. The study was conducted by the National Center for the Research and Rehabilitation of the Hereditary Ataxias in the city of Holguin (CIRAH), which is the main referral center for these conditions in the country.

### Patients

One hundred and thirty-six patients (61 female, 75 male) affected with autosomal dominant cerebellar ataxia without the abnormal expansion in the *ATXN2* gene, were tested for *ATXN3* mutation in the CIRAH. Also 65 healthy subjects (31 female; 34 male) were included as control group. The study was approved by the Institutional Ethics Committee. Written informed consent was obtained in all cases.

### Neurological and genealogical assessments

The neurological examination for all subjects was performed following Mayo Clinic procedures [36], also the genealogical information was obtained throughout a standardized interview.

### Molecular genetics studies

Genomic DNA was isolated from peripheral blood leucocytes using a standard protocol [37]. The *ATXN3* CAG repeat was assessed by polymerase chain reaction (PCR) amplification with the previously published MJD52 and MJD25 oligonucleotide primers [38] and fragment analysis using ReproGel™ high resolution in an ALFexpress sequencer (Amersham Biosciences, Sweden).

### Statistical analysis

Statistical analysis was conducted using the STATISTICA data analysis software system, version 6. StatSoft, Inc., 2003 (www.statsoft.com). The normality of all variables was assessed through the Kolmogorov-Smirnov (K-S) test. Mean comparisons were performed using the Student T-test. Correlation was assessed by the Pearson test. Statistical significance was considered when p < 0.05.
